# Osseodensification: An Innovative Technique With Manifold Gains

**DOI:** 10.7759/cureus.60255

**Published:** 2024-05-14

**Authors:** Sheetal R Khubchandani, Trupti Dahane, Surekha A Dubey

**Affiliations:** 1 Prosthodontics, Sharad Pawar Dental College and Hospital, Datta Meghe Institute of Higher Education and Research, Wardha, IND

**Keywords:** osseodensification, secondary implant stability, primary implant stability, osseointegration, implant therapy

## Abstract

Prosthodontics, which is removable and fixed, is the branch dealing with the replacement of missing teeth. Implant therapy is the popular treatment modality and commonly preferred treatment option by many patients and clinicians for missing teeth in recent years. Primary implant stability (PIS) is one of the crucial factors for osseointegration. It has been considered a crucial factor in the success of implants. Moreover, several factors influence PIS. On the other hand, both secondary implant stability and osseointegration are influenced by the PIS. Bone density, bone volume, bone-to-implant contact, and other factors that enhance or degrade the primary stability. Certain host sites such as the maxillary posterior region demand more dense bone to achieve desired results as they are the low-density areas of the jaw. So, a new promising and growing innovative concept of osseodensification (OD) offers a great solution with multiple benefits and desirable results. This review article aims to enlighten the multiple benefits of OD technique and their mechanism of action.

## Introduction and background

Prosthodontics is the branch that deals with two types of prostheses, namely removable and fixed. Fixed prostheses include conventional fixed dental prostheses (FDPs) and crowns. Additionally, dental implants are considered a major part of fixed prosthodontics. Dental implants are widely used nowadays to replace missing teeth and are routinely practiced and preferred by general dentists, prosthodontics, and oral and maxillofacial surgeons. It can be widely used, ranging from a single implant to full-mouth rehabilitation. Though implant therapy shows higher success rates, in some areas of the jaw, the success rate of dental implants is variable. For example, low-density areas, such as the maxillary posterior region, show variable results with implant therapy. The outcome of implant therapy is very dependent on its primary stability. It is provided by the mechanical contact between the external surface of the implant and the inner surface of the osteotomy [1].

The glossary of prosthodontic terms (GPT)-09 defines primary stability as nothing but contributing factors to the mechanical stabilization of a dental implant during the healing phase [2]. The healing process and success of dental implants are directly related to factors such as primary and secondary implant stability. In cases such as immediate implant placement after extraction or immediate loading of the implant on the day of placement, it is of prime significance. Total stability is measured by a combination of two phases: primary stability, which is achieved during placement itself, and secondary stability, which is measured after complete healing and radiographic evidence of osseointegration [3]. Secondary stability is dependent on primary stability, and thus it leads to a healthy and faster osseointegration, reducing the chances of implant failure.

The posterior maxilla is known to have low bone density. After osteotomy, a certain amount of bone is further lost while preparing the place for implants. This leads to an insufficient amount of bone around the external surface of implants. This can adversely affect the histo-morphometric parameters. It includes percentage bone-to-implant contact (%BIC) and percentage bone volume (%BV) and consequently, both stabilities are affected. Also, all these parameters affect the insertion and removal torque and the micromotion of the implants [4-6]. Ottoni et al. conducted a study in which they placed single-tooth implants and restored them. Their study demonstrated a failure reduction rate of 20% in single-tooth implant restoration when 9.8 Ncm of torque was added. So, they concluded that as the insertion torque is increased, it leads to fewer failures [7].

There are many techniques or modifications that are suggested in conventional drilling so as to improve the quality of osseointegration. One of the conventional techniques includes the preparation of the implant site using an undersized bur so that an undersized preparation is formed [8,9]. Also, some authors advocated the use of certain instruments called osteotomes [10,11]. Additionally, some authors advised the use of specifically designed implants for low-density bone. The use of mini implants and short implants is also advocated, depending on the available bone height and width at the site of implant placement [12]. All these techniques were used to improve the quality of treatment by using conventional implants and conventional techniques. However, they also come with certain disadvantages, as outlined below. Osteotomes allow the fracturing of trabeculae and engage in osteotomy, but it does not significantly improve other parameters such as percent volume [13,14]. It was also observed by clinicians that fractured bone trabeculae also hamper the healing process when compared to conventional drilling procedures [15].

Various other factors, such as decreasing bone density with advancing age. Especially in females with menopause or diseases such as osteoporosis, there are increased chances of diminished bone quality and quantity. After extraction of the tooth, maximum resorption takes place in the first year about 25% of alveolar bone is lost [16,17]. Surgical or pre-prosthetic procedures to improve bone quality and quantity before implant placement include (a) Horizontal and vertical augmentation of bone, in which bone is augmented in the form of graft material at the site of implant placement. It can be done in the same surgical visit or an additional visit can be required and post healing implant can be placed; (b) Guided bone regeneration is a technique, in which barrier membranes are used to direct the growth of bone; (c) Osseodistraction osteogenesis is a technique where the bone is cut, and a distractor is placed to pull both parts apart slowly and promote bone formation. This technique is very well utilized for other bones of the body as well in orthopedics; (d) The ridge-splitting technique is used when the bone is narrow. It is also called as ridge expansion and increases the width of the ridge [18-21]. All the above-mentioned techniques have variable results with less complication. The limitation of all the procedures is that they require additional surgical sitting. Patient compliance is needed for the additional surgical procedure. So, an insight to overcome all these drawbacks led to the emergence of a new technique called osseodensification (OD).

## Review

OD is a novel, innovative technique, invented in 2013 by Dr. Salah Huwais. This technique includes biomechanical bone preparation or osteotomy preparation performed for dental implant placement, utilizing a specialized set of burs known as the Densah Bur Kit (Versah-The Osseodensification Company, Jackson, USA). Conventional burs, which are used for implant placement, create subtraction drilling or osteotomy. In this technique, bone is removed while drilling. The Densah Burs are designed to form an osteotomy that enhances primary stability through a process known as non-subtractive drilling, non-extraction, or non-excavation drilling. So the bone is not removed and preserved there. These burs combine the advantages of osteotomes with the speed and tactile control of the drills during osteotomy [22].

Characteristics of the bur

The specialized burs used for the OD have the following features. The body of the bur is conical in shape and tapered. The shank is differently designed. It has a larger diameter at the proximal end, while it becomes smaller towards the apex or apical end. This end includes a minimum of one lip. The bur can rotate in two directions. When the bur rotates counterclockwise, it grinds the bone. This is termed the non-cutting process or the burnishing process. The bone is grinded but not removed. It grinds and re-deposits it there, giving a burnishing effect. When the bur rotates clockwise, it cuts the bone. This is termed the cutting process or the drilling process. The flutes of the bur are helical, and each flute has two faces. One face is cutting and another one is for burnishing. The action depends on the direction of rotation, whether it is clockwise or counterclockwise. The continuous rotation of the bur for a longer period of time causes the generation of force, and thereby, a push-back phenomenon occurs. It provides the operator with better control over expansion.

Mechanisms of the action

A standard surgical engine rotating at 800-1200 rpm can be used along with densifying burs. This is an additional advantage because the same engine can be used for both conventional and OD burs. The action of the bur, such as cutting or non-cutting, depends on the direction of the clockwise or anti-clockwise rotation, respectively [23].

Advantages of the technique

The preservation of the bone is the best advantage of this technique over conventional. There is no excavation of the bone that results in grafting of the bone automatically, with fewer traumas, when the bur is rotated anticlockwise. The condensed autograft is created by this procedure. The bone, which is cut and then redeposit in the peripheral walls of the osteotomy, is termed a condensed autograft [24]. A circumferential osteotomy with utmost precision is created, with a very small diameter. It is about 0.5 mm smaller than the conventional one. There's no need to use smaller diameter burs, as conventionally done. So the precision and accuracy of the surgery are maintained, and more predictable results can be expected. The generation of heat is remarkably decreased with surplus saline irrigation, along with a bouncing-pumping motion of bur. This leads to reduced necrosis and faster healing. As the implant surface is coated with the condensed autograft, healing is better and faster as compared to conventional drilling. The insertion torque and removal torque, %BV, and %BIC are higher compared to traditional drilling techniques. The volume increases as the cut bone is re-deposited there, thereby improving all other parameters.

Discussion

Lahens et al. conducted an in-vivo animal study. He concluded that insertion torque is increased when the OD procedure is used for implant placement than conventional drilling. As insertion torque is directly related to primary stability, it also improves primary implant stability (PIS) [25]. Slete et al. conducted a study to compare parameters after conventional drilling procedures and OD. He stated that there is a 60% increase in bone-to-implant contact by using OD, and this is four times more than the BIC achieved in conventional drilling procedures [26]. Also, studies confirmed a positive correlation between insertion torque and BIC. Thus, an increase or decrease in one parameter is directly proportional to another [27].

Normally, to achieve secondary stability it takes eight to 12 weeks after implant placement. Pai et al. stated that this time duration to achieve stability also decreases with the OD technique. Further, the reason for this is the reduced time taken for the remodeling of the bone [1]. In their study, Trisi et al. added an advantage of this technique i.e. ridge expansion. He stated that due to ridge expansion by this technique, it can be used where available bone width is less or unfavorable for implant placement. Conventionally, ridge splitting is performed in narrow bone areas. So, the OD proves helpful in those areas as well by avoiding the risk of complications of ridge splitting [4].

With all the advantages mentioned above, this technique is a boon to implant dentistry. This technique can be applied to achieve excellent treatment output with immediate or early loading of implants as well [22]. The systematic review by Fontes Pereira et al. literature shows that the OD method improves PIS, %BIC, and clinical outcome in low-density bone (type IV). Such bone is commonly seen in maxillary posterior areas. Furthermore, post-extraction implants, narrow alveolar ridge expansion, and maxillary sinus elevation may be possible with this method. This is also possible with conventional technique, but additional equipment and sometimes bone grafting are also required. However, OD itself can provide autograft. Nevertheless, given the limitations and biases of the studies, care should be taken when interpreting these conclusions. Consequently, additional research demonstrating increased methodological rigor and external validity is required to validate the advantages of the OD methodology in oral implantology [28].

Whether the OD promotes implant primary stability is still up for debate, given the scant information on the osteocompaction and OD approach, which is limited to animal studies with significant risk of bias, as well as case reports and case series. To provide evidence-based recommendations, well-designed in vivo animal and human studies with extended follow-up times are needed. There aren't many studies on OD in the literature; the majority are case reports with little to no supporting evidence and animal experiments. While the OD appears to be a potential method for enhancing bone quantity and quality, the results are conflicting and need to be considered cautiously. Before implementing such a strategy into routine everyday practice, well-designed in vivo animal and human trials with longer follow-up periods are needed [29].

## Conclusions

Though implant therapy is routinely practiced, still some patients deny implant therapy due to the involved steps and time factors. They demand a definitive treatment in less time. With the invention of specialized OD burs for osteotomy, treatment time has reduced, and also implant therapy outcomes have become predictable. The current literature evidence, though not very long-term, has shown very good results as far as the quality of final osseointegration is concerned. Hence, this technique is here to stay. However, long-term prospective trials, especially in the maxillary posterior region, are required. The results are conflicting and should be viewed with caution, despite the fact that the OD appears to be a very promising approach. It is necessary to conduct carefully planned research with extended follow-up times on both humans and animals before incorporating such a technique into routine everyday practice.
